# Novel Penicillin-Type Analogues Bearing a Variable Substituted 2-Azetidinone Ring at Position 6: Synthesis and Biological Evaluation

**DOI:** 10.3390/molecules201219828

**Published:** 2015-12-10

**Authors:** Margherita De Rosa, Giovanni Vigliotta, Giuseppe Palma, Carmela Saturnino, Annunziata Soriente

**Affiliations:** 1 Dipartimento di Chimica e Biologia, Università degli Studi di Salerno, via Giovanni Paolo II, 132, Fisciano 84084, Italy; titti@unisa.it; 2 S.S.D. “Sperimentazione Animale”, National Cancer Institute, I.R.C.C.S. “Fondazione Pascale”, Naples 80131, Italy; palma.giuseppe@icloud.com; 3 Dipartimento di Farmacia, Università degli Studi di Salerno, via Giovanni Paolo II, 132, Fisciano 84084, Italy; saturnino@unisa.it

**Keywords:** 6-aminopenicillanic acid (6-APA), 2-azetidinone, β-lactam antibiotics, antibacterial, Staudinger reaction

## Abstract

The synthesis and the biological activity of novel semi-synthetic β-lactam compounds containing an azetidinone moiety joined to the amino-nitrogen of the (+)-6-aminopenicillanic acid (6-APA) as new antibacterial agents is reported. The synthesized compounds were screened for their *in vitro* antimicrobial activity against a panel of Gram positive and Gram negative pathogens and environmental bacteria. Tested compounds displayed good antimicrobial activity against all tested Gram positive bacteria and for *Staphylococcus aureus* and *Staphylococcus epidermidis* antimicrobial activity resulted higher than that of the reference antibiotic. Additionally, *in vitro* cytotoxic screening was also carried out indicating that the compounds do not cause a cell vitality reduction effective at concentration next to and above those shown to be antimicrobial.

## 1. Introduction

**Figure 1:**
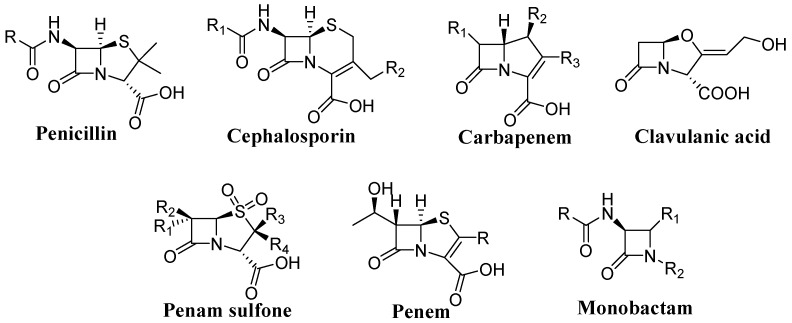
Representative β-lactam structures.

Since the discovery of penicillin, and shortly there after cephalosporin, the key role of β-lactam framework was recognized thus catching the attention of medicinal researchers and organic chemists. The β-lactam nucleus is the structural feature and the core of the biological activity of one of the most successful classes of therapeutic agents to date characterized by a broad spectrum of activity and low toxicity (Figure 1) [1,2,3,4,5,6,7,8,9]. Unfortunately, long-term use related to the overuse and misuse of β-lactam antibiotics have resulted in the proliferation of resistant organisms among a variety of clinically significant species of bacteria becoming an important worldwide problem. Their effectiveness has been seriously compromised by the bacterial ability to develop different competitive mechanisms in order to survive [10,11,12,13,14]. In particular, resistance to β-lactams in many bacteria is usually due to the production of β-lactamases [15,16,17,18,19], enzymes that inactivate the drug by hydrolysis of the β-lactam ring preventing the action against its original cellular targets, or the modification of penicillin-binding proteins (PBPs) or cellular permeability. In response to this challenge, substantial research is devoted to the design and synthesis of new classes of antimicrobial agents with greater potency, stability and efficacy than existing drugs. In fact, attention in this field has not yet been exhausted because the basic difficulty is the rapid development of drug resistant pathogenic bacteria.

In line with this rationale and as a part of our ongoing study on the synthesis of antimicrobial active molecules [20], we have planned a synthetic strategy to synthesize a set of new penicillanic acid (6-APA) derivatives containing an additional appropriately functionalized β-lactam nucleus joined to the amino-nitrogen of 6-APA with the hypothesis that a synergistic effect should be the result of the presence of the additional 2-azetidinone ring on the 6-APA scaffold thus enhancing the drug stability and biological activity. The synthesis and characterization of a number of new penicillanic acid derivatives is described and their biological activities are screened.

## 2. Results and Discussion

### 2.1. Chemistry

**Scheme 1:**
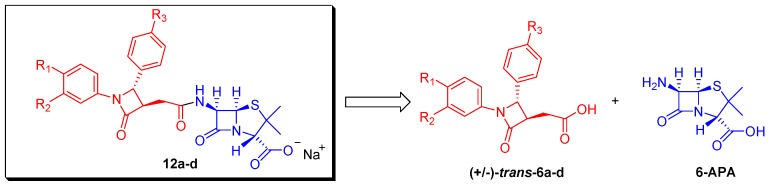
General retrosynthetic strategy.

Scheme 1 shows the retrosynthetic analysis of the penicillanic acid derivatives **12a**–**d**. The construction of **12** is implemented with a simple strategy based on the coupling of the 6-APA nucleus with a functionalized 2-azetidinone ring (**6a**–**d**) built through [2+2] cycloaddition reaction of a ketene with an imine (Staudinger reaction) [21,22,23,24]. The presence of an acid functionality in C-3 position of the β-lactam derivatives **6a**–**d** directly engages the β-lactam unit with 6-APA nucleus by standard amide coupling methodology and provides an amide functionality in the connecting linker directly attached at position 6 of 6-APA, a structural feature common to various bio- and semi-synthetic penicillins [25].

It is reported that the presence of a phenyl group at the C-4 position of the azetidinone ring benefits a hydrophobic interaction with the active site of beta-lactamases, thus increasing the ability of the molecules to inhibit them [1]. Structure-activity studies show the significant influence on the biological activity exerted also by the presence of a phenyl group at the N-1 position and of the different substituents on the N-1 aromatic ring [3,4,26]. Therefore, keeping these studies in view, we decided to combine 1,4-diaryl-2-azetidinones with 6-APA in search of a cooperative effect on the biological activity, possibly resulting in an improvement of antibacterial activity, and our hypothesis is confirmed by the biological data.

**Scheme 2:**
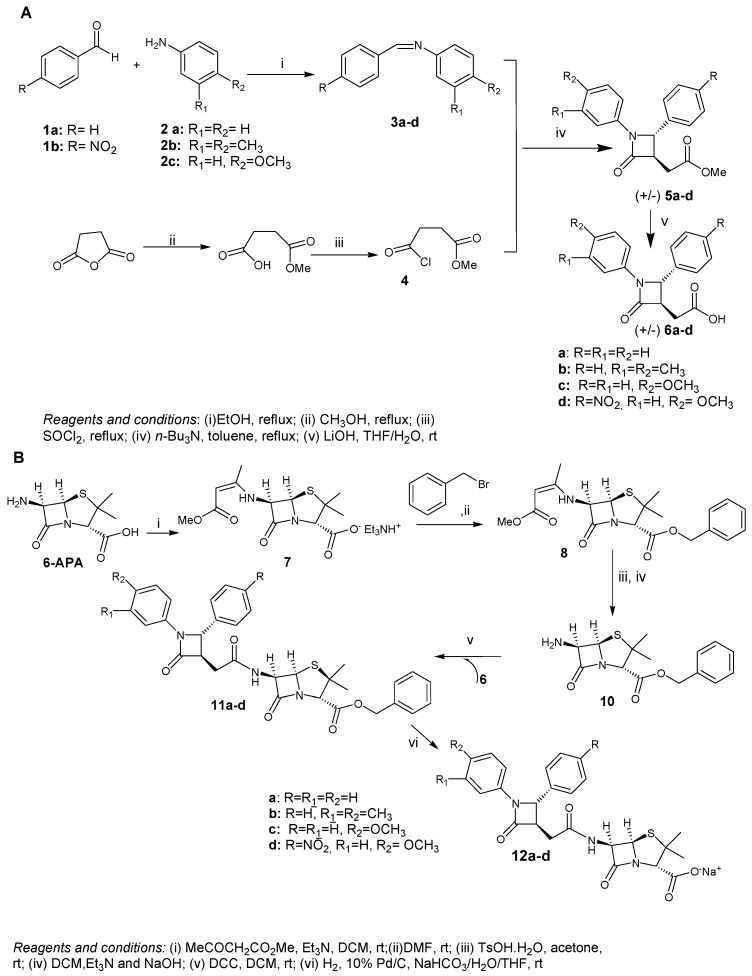
(**A**) General synthetic route to compounds **5a**–**d**; (**B**) General synthetic route to compounds **12a**–**d**.

The target compounds **12a**–**d** were synthesized as depicted in Scheme 2. The preparation of 2-azetidinones **5a**–**d** (Scheme 2A) is performed by the Staudinger [2+2] cycloaddition reaction between properly substituted imines **3** and a ketene prepared *in situ* from methyl 3-(chloroformyl)propionate **4** [24,27,28]. The preparation of the imine precursor **3a**–**d** was achieved in good yields (85%–96%) by condensation of the properly substituted aldehydes and anilines in ethanol [29,30,31,32]. The Staudinger reaction is one of the most widely employed methods in the preparation of β-lactam nucleus because it provides direct access to variously functionalized 2-azetidinones and allows to modulate the stereochemical outcome of the reaction. Under our experimental conditions, we obtained *trans*-**5**-cycloadduct only in moderate yield (45%–60%). The stereoselectivity of this reaction was unambiguously assigned by ^1^H-NMR coupling constant analysis regarding the protons at C3 and C4 in accordance with literature data for *trans*-β-lactam [21]. With the aim of increasing the reaction efficiency, different protocols were used, changing solvent, temperature and order of addition of the reagents, but we did not obtain any improvement.

Next, the hydrolysis of **5** with LiOH solution afforded corresponding acid **6** in almost quantitative yield [24]. This intermediate was then directly used for the coupling reaction with 6-amino penicillanic nucleus. At first, we tried to perform the direct coupling of **6** with the commercially available and inexpensive (+)-6-APA, but the formation of a complex mixture of products was observed. Therefore, we considered a different strategy based on the coupling of azetidinone key intermediate **6** to the benzyloxy ester of (+)-6-APA [33], followed by hydrogenolysis in a mixture of THF/aqueous NaHCO_3_ to deprotect the benzyl ester and directly furnish the target compounds as sodium salt (Scheme 2B) [34]. (+)-6-APA was *N*-protected using ethyl acetoacetate, then esterified with benzyl bromide and next the amine function of (+)-6-APA ester was liberated by treatment with *p*-toluensulfonic acid affording **10** [33]. The free amine **10** was so added to a preformed mixture of acid **6** and DCC in order to afford compound **11** as mixture of diastereomers, with the 2-azetidinone ring directly coniugated to 6-APA residue [33]. Thus, derivatization of 6-APA led to higher coupling reaction yields and easier isolation of target compounds. Finally, to ensure water-solubility of the final compounds hydrogenation of **11** over Pd/C (10%) in THF/aqueous NaHCO_3_ at room temperature afforded the target molecules **12** in high yield [34]. All the reported compounds were characterized by analytical spectroscopic methods (^1^H- and ^13^C-NMR, MS) and elemental analysis.

### 2.2. Biological Work

#### 2.2.1. Antimicrobial Activity of Compounds **12a**–**d**

The antibacterial activity of new compounds was evaluated by determining the minimum inhibitory concentration (MIC), according to the CLSI (Clinical and Laboratory Standards Institute, formerly the NCCLS) guidelines and minimum lethal dose (MLD) [35]. All the synthesized compounds **12a**–**d** were tested against pathogenic and non-pathogenic strains, representative of Gram positive (*Staphylococcus aureus*, *Staphylococcus epidermidis*, *Bacillus* sp.) and Gram negative bacteria (*Escherichia coli*, *Salmonella entericaserovar* Typhimurium, *Pseudomonas fluorescens* and *Pseudomonas aeruginosa*). Ampicillin was used as the reference compound, a derivative of the 6-aminopenicillanic acid (6-APA), clinically used for the treatment of infections caused by Gram positive (*Streptococcus* spp., *Staphyloccus* spp.) and few Gram negative bacteria (*Neisseria*, some *Enterobacteriaceae*).

Table 1 shows that four compounds, on the whole, did not exhibit antimicrobial activity towards Gram negative considered, although the analysis of MIC_50_ evidenced that **12a** showed some growth inhibition activity against *Salmonella* (MIC_50_ > 64 μg·mL^−1^). In contrast, they were very effective against all examined Gram positive (Table 1 and Table 2). In order to better interpret the results, MIC and MLD were expressed also in terms of molarity (values in parentheses in Table 1 and Table 2). The MIC_100_ and/or MLD analysis indicated that all four modifications of the 6-APA improved the antimicrobial activity against *S. aureus*, compared to ampicillin. The compound most efficient was **12a**, with both the MIC_100_ and MLD two times as massic concentration and almost three times lower molar concentration than that of ampicillin (Table 2). Similar results were obtained against *S. epidermidis*, but in this case improvements were more evident by comparing the molar concentrations. For this bacterial strain, the derivative **12a** turned out the most active in terms of MIC, two times lower than MIC of ampicillin as molar concentration, while the **12c** presented the highest biocidal action (Table 1 and Table 2). All new compounds were very effective also against the *Bacillus* strain, although by MIC and MLD analysis there were no obvious improvements compared to the activity of ampicillin. Among the four new compounds, the highest antimicrobial activity was observed for **12a** and **12b**, the first with the lowest values of MIC and MLD (Table 1 and Table 2).

Thus, all synthesized compounds were characterized by a strong antimicrobial action towards Gram positive, and as reported in Table 2 (column MIC_a–d_/MIC_a_) **12a** was the one with greater activity. In addition, **12a** was the only compound with a slight activity also against Gram negative, confirming its best antibacterial ability.

**Table 1:** Minimal inhibitory concentration (MIC) and Minimal lethal dose (MLD) as µg·mL^−1^. The numbers in parentheses indicate the molar concentration (μM).

Compd.	*Escherichia coli*		*Salmonella typhimurium*		*Pseudomonas* spp. (*fluorescens, aeruginosa*)		*Staphylococcus aureus*		*Staphylococcus epidermidis*		*Bacillus* sp.	
	MIC_100_	MLD	MIC_100_	MLD	MIC_100_	MLD	MIC_100_	MLD	MIC_100_	MLD	MIC_100_	MLD
**Amp**	48	≤256	8.0	≤256	>256	>256	2.0 (5.8)	8.0 (23.2)	0.35 (1.0)	16.0 (46.0)	0.25 (0.72)	8.0 (23.2)
**12a**	>256	>256	>256	>256	>256	>256	1.0 (2.1)	4.0 (8,4)	0.25 (0.52)	16.0 (33.6)	0.5 (1.05)	32.0 (67.2)
**12b**	>256	>256	>256	>256	>256	>256	<1.5(3.9)	32.0 (63.4)	0.35 (0.69)	≥16.0 (≥31.7)	>1.0; <1.5 (>1.98; <2.97)	48.0 (86.4)
**12c**	>256	>256	>256	>256	>256	>256	>2.0; <3.0 (>3.9; <5.9)	<4.0 (<7.9)	<0.35 (<0.69)	8.0(15.8)	2.0(3.94)	128.0 (252.2)
**12d**	>256	>256	>256	>256	>256	>256	1.75 (3.45)	32.0 (57.6)	0.75 (1.35)	≥16.0 (≥28.8)	2.0 (3.6)	64.0 (115.2)

MIC_100_ = Minimal compound concentration that completely inhibits bacterial growth; Amp = ampicillin. Each value represents the mean of three independent determinations.

**Table 2:** Antimicrobial efficiency of compounds evaluated by MICs and MLDs ratios. In parentheses are indicate the ratios of the MIC expressed as molarity (μM). Each value was obtained from the ratio of MIC_100_ indicated in Table 1.

Compd.	*S. aureus*			*S. epidermidis*			*Bacillus* sp.		
	MIC_Cmp_/MIC_Amp_	MLD_Cmp_/MLD_Amp_	MIC_a–d_/MIC_a_	MIC_Cmp_/MIC_Amp_	MLD_Cmp_/MLD_Amp_	MIC_a–d/_MIC_a_	MIC_Cmp_/MIC_Amp_	MLD_Cmp_/MLD_Amp_	MIC_a–d/_MIC_a_
**12a**	0.5 (0.36)	0.5 (0.36)	1	0.71 (0.52)	1.0 (0.73)	1	2.0 (1.46)	4.0 (2.89)	1
**12b**	<0.75 (<0.67)	4.0 (2.73)	<1.5 (<1.86)	1.0 (0.69)	≥1.0 (≥0.69)	1.4 (1.33)	>4.0, <6.0 (>2.75, <4.12)	6.0 (3.72)	>2.0, <3.0 (>1.88, <2.83)
**12c**	>1.0, <1.5 (>0,67, <1.01)	<0.5 (<0.34)	>2.0, <3.0 (>1.87, <2.81)	<1.0 (<0.69)	0.5 (0.34)	<1.4 (<1.33)	8.0 (5.47)	16.0 (10.87)	4.0 (3.75)
**12d**	0.87 (0.6)	4.0 (2.48)	1.75 (1.64)	2.14 (1.35)	≥1.0 (≥0.63)	2.14 (2.6)	8.0 (5.0)	8.0 (4.96)	4.0 (3.43)

MIC_Cmp_ = MIC of indicated compounds (**a**–**d**); MIC_Amp_ = MIC ampicillin; MIC_a–d_ = MIC of compounds **12a**, **b**, **c**, **d**; MIC_a_ = MIC of compound **12a**; MLD_Cmp_ = MLD of indicated compounds (**a**–**d**); MLD_Amp_ = MLD of ampicillin.

#### 2.2.2. Cytotoxicity Assays

**Figure 2:**
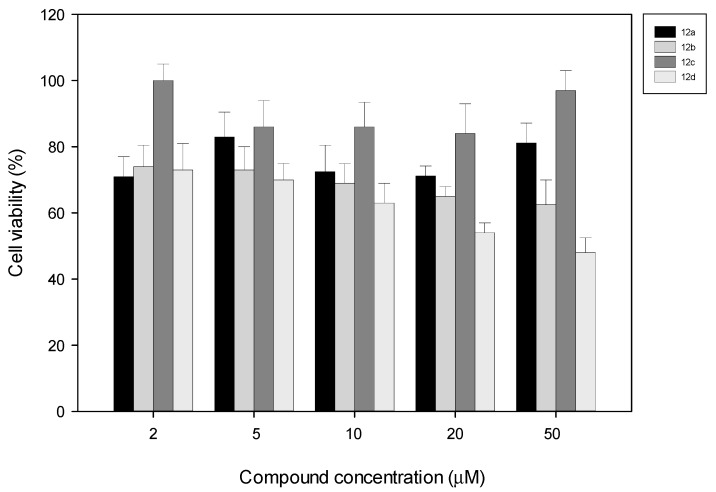
Effect of synthesized compounds on cell viability. NIH-3T3 cells were exposed 72 h at indicated concentration of **12a**, **b**, **c**, **d**. Inhibition activity of compounds was expressed as percentages of control (0.5% DMSO). Each point represents the mean ± SD of the six experiments.

*In vitro* cytotoxicity of the new compounds was carried out evaluating viability of NIH-3T3 cells after 72 h of incubation in the presence of increasing concentrations of antibiotics (2–50 μM) (Figure 2). Cell viability was assessed by using the MTT assay as described in the Experimental Section. As shown in Figure 2, the presence of **12a**, **12b** and **12d** induced a mild reduction of vitality respect to the control (0.5% DMSO alone), however for **12d** higher values (10–50 μM) a dose-dependent reduction of cell viability was observed indicating a potential toxicity. Compound **12c** showed slight or no cytotoxic effect on the cell line at concentration between 2 and 50 μM. Thus, these results suggest that tested compounds do not show *in vitro* relevant effects on the cell viability at concentration tens of times higher than MICs found in examined Gram-positive bacteria (see Table 1)*.*

## 3. Experimental Section

### 3.1. Chemistry

#### 3.1.1. Material and Methods

All reagents and anhydrous solvents were obtained from commercial sources and used without further purification. All reactions requiring anhydrous conditions were performed under N_2_ atmosphere and all glassware were flame dried. Elemental analyses were performed on the FlashEA 1112 Series with Thermal Conductivity Detector (Thermo Scientific Corporation, Waltham, MA, USA). ^1^H- and ^13^C-NMR spectra were recorded on Bruker (Rheinstetten, Germany) DRX 600, 400, 300, 250 spectrometers (600 MHz, 400 MHz , 300 MHz, 250 MHz for ^1^H; 151 MHz, 100 MHz, 75 MHz, 62.89 MHz for ^13^C. *J* values are given in Hz. The ^1^H chemical shifts were referenced to the solvent peak: CDCl_3_ (7.26 ppm), and the ^13^C chemical shifts were referenced to the solvent peak: CDCl_3_ (77.0 ppm). For D_2_O, ^13^C-NMR spectra were referenced to the signal for the carbonyl group of acetone (one drop, added as an internal standard), which was set to 215.94 ppm. ESI(+)-MS measurements were performed on a Waters 4 micro quadrupole mass spectrometer equipped with electrospray ion source (Waters, Milford, MA, USA). IR spectra were recorded on an FT-IR instrument Bruker Vector 22 (Bruker, Ettlingen, Germany). Melting points were performed on DSC 2920 TA INSTRUMENTS (TA Instruments-Division of Waters, Milano, Italy). Thin-layer chromatography was performed on Macherey-Nagel (Macherey-Nagel GmbH & Company, Düren, Germany) pre-coated aluminum sheets (0.20 mm, silica gel 60 with fluorescent indicator UV_254_) in appropriate solvent. Column chromatography was carried out using silica gel 60 (70–230 mesh ASTM, Merck, Darmastdt, Germany).

Compounds **3a**–**d**were prepared according to the previously described procedure and their spectral features matched with those reported in literature [29,30,31,32].

#### 3.1.2. Synthesis of Compounds **3a**–**d**. General Method

The aldehyde 1 (11 mmol) was added to a solution of anilines 2 (11 mmol) in absolute ethanol (40 mL). The resulting mixture was heated at reflux for 4 h and left standing overnight at room temperature. After the reaction was complete, the solution was cooled and the precipitate formed was collected by filtration. The product was washed with cold water and purified by recrystallization.

*N-Benzylidenebenzenamine* (**3a**). Recrystallized from ethanol as pale colorless needles; m.p. 50–51 °C. ^1^H-NMR (300 MHz, CDCl_3_): δ 7.25–7.28 (m, 3H), 7.44 (t, *J* = 8.3, 2H), 7.50–7.54 (m, 3H), 7.94–7.97 (m, 2H), 8.49 (s, 1H); ^13^C-NMR (75 MHz, CDCl_3_): δ 120.8, 125.9, 128.7, 128.8, 129.1, 131.3, 136.2, 152.0, 160.3 [29,30].

*N-Benzylidene-3,4-dimethylbenzenamine* (**3b**). Recrystallized from ethanol as yellow solid [31]; m.p. 42–43 °C. ^1^H-NMR (400 MHz, CDCl_3_): δ = 2.28 (s, 3H), 2.30 (s, 3H), 6.99–7.00 (m, 1H), 7.05 (s, 1H), 7.15 (d, *J* = 7.9, 1H), 7.46–7.48 (m, 3H), 7.88–7.91 (m, 2H), 8.47 (s, 1H); ^13^C-NMR (100 MHz, CDCl_3_): δ = 19.2, 19.7, 117.9, 122.2, 128.6 (×2), 130.1, 131.0, 134.4, 136.2, 137.2, 149.7, 159.3.

*N-Benzylidene-(4-methoxybenzenamine)* (**3c**). The reaction was performed at room temperature affording the title compound after 3 h [30]. Recrystallized from ethanol as gray plates; m.p. 71*–*72 °C. ^1^H-NMR (300 MHz, CDCl_3_): δ = 3.83 (s, 3H), 6.93 (d, *J* = 8.4, 2H), 7.23 (d, *J* = 8.4, 2H), 7.43–7.47 (m, 2H), 7.86–7.92 (m, 2H), 8.48 (s, 1H); ^13^C-NMR (100 MHz, CDCl_3_): δ = 55.4, 114.2, 122.0, 128.4, 128.5, 130.8, 136.3, 144.7, 158.1, 158.2.

*N-(4-Nitrobenzylidene)-4-methoxybenzenamine* (**3d**). Recrystallized from ethanol as yellow solid [32]; m.p. 126–128 °C. ^1^H-NMR (400 MHz, CDCl_3_): δ = 3.85 (s, 3H), 6.96 (d, *J* = 8.8, 2H), 7.31 (d, *J* = 8.8, 2H), 8.06 (d, *J* = 8.4, 2H), 8.31 (d, *J* = 8.4, 2H), 8.58 (s, 1H); ^13^C-NMR (100 MHz, CDCl_3_): δ = 55.4, 114.20, 122.1, 128.6, 130.9, 136.4, 142.4, 144.6, 150.6, 158.2.

#### 3.1.3. Methyl 4-chloro-4-oxobutanoate (**4**).

The product was prepared as a pale yellow oil from succinic anhydride according to the literature procedure in 98% yield and was used without further purification [27,28].

^1^H-NMR (300 MHz, CDCl_3_): δ = 2.68 (t, *J* = 6.4, 2H), 3.21 (t, *J* = 3.4, 2H), 3.71 (s, 3H); ^13^C-NMR (75 MHz, CDCl_3_): δ = 173.0, 171.2, 52.1, 41.6, 29.0.

#### 3.1.4. General procedure for the synthesis of azetidin-2-ones **5a**–**d**

A flame-dried round-bottom flask was charged with a solution of imine **3** (2 mmol) and *n*-tributylamine (6 mmol, 3 equiv.) in dry toluene (8 mL). The solution was heated to reflux and the acyl chloride **4** (4 mmol, 2 equiv.) was then added dropwise through a syringe. The resulting solution was kept at reflux temperature for 22 h. After cooling, 1M HCl was added and the mixture was stirred for an additional 15 min. The mixture was transferred to a separatory funnel and extracted with AcOEt (2 × 10 mL). The combined organic layers were washed first with saturated NaHCO_3_ aqueous solution (20 mL), brine (20 mL), and finally dried over Na_2_SO_4_, and evaporated. The oily crude mixture was purified by column chromatography (silica gel, 7:3 hexane/AcOEt) affording *trans*-2-azetidinone compound **5** [24].

*Methyl 2-(2-oxo-1,4-diphenylazetidin-3-yl)acetate* (**5a**). Brown solid (45% yield, m.p. 144–146 °C). IR (KBr pellet, cm^−1^): ν 2958, 1746, 1597, 1498, 1389; ^1^H-NMR (300 MHz, CDCl_3_): δ 2.83 (dd, *J* = 10.2, 16.7, 1H), 2.99 (dd, *J* = 4.7, 16.1, 1H), 3.36–3.42 (m, 1H), 3.70 (s, 3H), 4.85 (d, *J* = 2.3, 1H), 6.91–7.06 (m, 2H), 7.14–7.38 (m, 8 H); ^13^C-NMR (75 MHz, CDCl_3_): δ 172.2, 166.0, 140.1, 139.9, 130.4 (×2), 128.0, 127.1, 126.3 (×2), 125.8 (×2), 125.2 (×2), 61.8, 55.1, 52.1, 32.5. MS: *m*/*z* 295 (M^+^). Anal. calcd. for C_18_H_17_NO_3_: C, 73.20; H, 5.80; N, 4.74%; found: C, 73.16; H, 5.83, N, 4.64 %.

*Methyl 2-(1-(3,4-dimethylphenyl)-2-oxo-4-phenylazetidin-3-yl)acetate* (**5b**). Colorless oil (60% yield). IR (KBr pellet, cm^−1^): ν 2950, 1736, 1612, 1513, 1315; ^1^H-NMR (400 MHz, CDCl_3_): δ 2.16 (s, 3H), 2.17 (s, 3H), 2.81 (dd, *J* = 10.2, 16.7, 1H), 2.97 (dd, *J* = 4.5, 16.7 , 1H), 3.32–3.37 (m, 1H), 3.69 (s, 3H), 4.81 (d, *J* = 2.3, 1H), 6.84–6.86 (m, 1H), 6.95–6.97 (m, 2H), 7.22–7.29 (m, 5H); ^13^C-NMR (75 MHz, CDCl_3_): δ 171.0, 165.7, 137.6, 135.3, 132.3, 129.9, 129.0 (×2), 128.4, 126.0 (×2), 118.6, 114.3, 61.0, 55.7, 52.1, 32.9, 29.7, 19.9, 19.3. MS: *m*/*z* 323 (M^+^). Anal. calcd. for C_20_H_21_NO_3_: C, 74.28; H, 6.55; N, 4.33%; found: C, 74.31; H, 6.53, N, 4.32%.

*Methyl-2-(1-(4-methoxyphenyl)-2-oxo-4-phenylazetidin-3-yl)acetate* (**5c**). Yellow solid (55% yield, m.p. 111–113 °C). IR (KBr pellet, cm^−1^): ν 2954, 1741, 1647, 1592, 1503; ^1^H-NMR (300 MHz, CDCl_3_): δ 2.82 (dd, *J* = 10.3, 16.7, 1H), 2.98 (dd, *J* = 4.6, 16.7, 1H), 3.31–3.38 (m, 1H), 3.69 (s, 3H), 3.73 (s, 3H), 4.81 (d, *J* = 2.1, 1H), 6.78 (d, *J* = 9.0, 2H), 7.22 (d, *J* = 9.0, 2H), 7.26–7.36 (m, 5H); ^13^C-NMR (75 MHz, CDCl_3_): δ 171.3, 156.1, 137.4, 131.1, 129.0 (×2), 128.5, 127.6, 126.1 (×2), 118.4 (×2), 114.3 (×2), 61.1, 55.7, 55.4, 52.0, 32.9. MS: *m*/*z* 325 (M^+^). Anal. calcd. for C_19_H_19_NO_4_: C, 70.14; H, 5.89; N, 4.31%; found: C, 70.16; H, 5.86; N, 4.30%.

*Methyl 2-(1-(4-methoxyphenyl)-2-(4-nitrophenyl)-4-oxoazetidin-3-yl)acetate* (**5d**). Brown solid (52% yield. m.p. 124–126 °C). IR (KBr pellet, cm^−1^): ν 2944, 1741, 1607, 1513, 1354; ^1^H-NMR (400 MHz, CDCl_3_): δ, 2.86 (dd, *J* = 11.2, 17.2, 1H), 3.04 (dd, *J* = 4.4, 17.2, 1H), 3.33–3.38 (m, 1H), 3.71 (s, 3H), 3.74 (s, 3H), 4.93 (d, *J* = 2.0, 1H), 6.79 (d, *J* = 9.2, 2H), 7.16 (d, *J* = 9.2, 2H), 7.57 (d, *J* = 8.8, 2H), 8.24 (d, *J* = 8.8, 2H); ^13^C-NMR (100 MHz, CDCl_3_): δ 171.2, 164.3, 156.5, 148.0, 144.9, 130.5, 127.2 (×2), 124.3 (×2), 118.3 (×2), 114.5 (×2), 60.3, 55.9, 55.4, 52.2, 32.8. MS: *m*/*z* 370 (M^+^). Anal. calcd. for C_19_H_18_N_2_O_6_: C, 61.62; H, 4.90; N, 7.56%; found: C, 61.65; H, 4.87, N, 7.58%.

#### 3.1.5. General Procedure for the Synthesis of **6a**–**d**

A 0.98M aqueous solution of LiOH (0.10 mL, 0.096 mmol) was added to a solution of *trans*-**5** (0.08 mmol) at room temperature in THF. The reaction mixture was stirred at room temperature for 5h and quenched with HCl 1 M (0.2 mL). The reaction mixture was diluted with ethyl acetate (1 mL) and washed with HCl 1M (1 mL), water (1 mL) and brine (1 mL). The combined organic layers were dried over magnesium sulfate and concentrated *in vacuo* to give crude acid **6** of at purity such as to be used without further purification [24].

*2-(2-Oxo-1.4-diphenylazetidin-3-yl)acetic acid* (**6a**). Brown solid (>99% yield). ^1^H-NMR (250 MHz, CDCl_3_): δ 2.89 (dd, *J* = 9.5, 17.0, 1H), 3.03 (dd, *J* = 5.2, 17.0, 1H), 3.38–3.49 (m, 1H), 4.84 (d, *J* = 2.5, 1H), 7.00–7.12 (m, 2H), 7.16–7.38 (m, 8H); ^13^C-NMR (75 MHz, CDCl_3_): δ 176.0, 165.6, 140.3, 139.9, 129.4 (×2), 129.1 (×2), 128.5 (×2), 126.3, 124.7, 122.0 (×2), 61.0, 55.2, 32.3.

*2-(1-(3.4-Dimethylphenyl)-2-oxo-4-phenylazetidin-3-yl)acetic acid* (**6b**). White solid (>99% yield). ^1^H-NMR (300 MHz, CDCl_3_): δ 2.16 (s, 3H), 2.17 (s, 3H), 2.86 (dd, *J* = 9.8, 17.0 Hz, 1H), 3.00 (dd, *J* = 5.0, 17.0 Hz, 1H), 3.34–3.40 (m, 1H), 3.69 (s, 3H), 4.81 (d, *J* = 2.2 Hz, 1H), 6.84–6.87 (m, 1H), 6.95–7.00 (m, 2H), 7.22–7.34 (m, 5H); ^13^C-NMR (75 MHz, CDCl_3_): δ 175.1, 165.8, 138.0, 137.4, 134.8, 132.5, 130.0, 128.9 (×2), 128.4, 126.1, 118.6 (×2), 114.5, 60.8, 55.2, 32.5, 19.3, 19.1.

*2-(1-(4-Methoxyphenyl)-2-oxo-4-phenylazetidin-3-yl)acetic acid* (**6c**). Brown oil (>99% yield). ^1^H-NMR (250 MHz, CDCl_3_): δ 2.02 (s, 3H), 2.84 (dd, *J* = 9.8, 17.0, 1H), 2.99 (dd, *J* = 5.0, 17.0, 1H), 3.29–3.39 (m, 1H), 3.70 (s, 3H), 4.80 (d, *J* = 2.25, 1H), 6.75 (d, *J* = 9.0, 2H), 7.19 (d, *J* = 9.0, 2H), 10.4 (bs, 1H); ^13^C-NMR (75 MHz, CDCl_3_): δ 175.5, 165.5, 156.2, 141.9, 137.1, 130.9, 129.1, 128.6, 126.1, 122.3, 118.5, 114.3, 61.1, 55.4, 55.3, 32.8.

*2-(1-(4-Methoxyphenyl)-2-(4-nitrophenyl)-4-oxoazetidin-3-yl)acetic acid* (**6d**). Brown oil (>99% yield). ^1^H-NMR (400 MHz, CDCl_3_): δ 2.92 (dd, *J* = 10.4, 17.6, 1H), 3.07 (d, *J* = 4.4, 17.6, 1H), 3.36–3.39 (m, 1H), 3.74 (s, 3H), 4.92 (d, *J* = 2.0, 1H), 6.79 (d, *J* = 9.2, 2H), 7.16 (d, *J* = 9.2, 2H), 7.54 (d, *J* = 8.8, 2H), 8.22 (d, *J* = 8.8, 2H); ^13^C-NMR (75 MHz, CDCl_3_): δ 175.0, 164.5, 156.5, 148.0, 144.6, 130.2, 127.1 (×2), 124.3 (×2), 118.3 (×2), 114. 5 (×2), 60.2, 55.4 (×2), 32.5.

#### 3.1.6. Synthesis of (2*S*,5*R*,6*R*)-Benzyl 6-amino-3,3-dimethyl-7-oxo-4-thia-1-aza-bicyclo[3.2.0]heptane-2-carboxylate **10**

A mixture of 6-APA (1g, 4.6 mmol) and triethylamine (1.30 mL, 9.2 mmol) in anhydrous dichloromethane (10 mL) was stirred at rt until dissolution before adding methyl acetoacetate (4.6 mmol, 0.502 mL). The stirring was continued for 3h and the solvent was then removed *in vacuo* affording **7**. To the crude **7** dissolved in DMF (10 mL) benzyl bromide (4.6 mmol, 0.54 mL) was added and the reaction mixture was stirred overnight. The solution was diluted with ethyl acetate and washed with brine (3 × 15 mL). The combined organic layers were dried over magnesium sulfate, filtered and concentrated under reduced pressure to give crude material **8** as a solid. A solution of **8** in acetone (10 mL) was treated with *p*-toluenesulfonic acid monohydrate (967 mg). The salt **9** precipitate after 10 min. was filtered and washed with ethyl ether (5 mL). Finally, crude **9** was solubilized with triethylamine (1.30 mL) in dichloromethane (50 mL) and stirred for 2 h. NaOH 1 M (10 mL) was added and the organic phase removed. The aqueous phase was extracted with dichloromethane and the resulting organic phase were combined, washed with brine (10 mL), dried and concentrated under vacuum to give **10** as yellow thick oil [33].

^1^H-NMR (300 MHz, CDCl_3_): δ 1.40 (s, 3H), 1.60 (s, 3H), 4.41 (s, 1H), 4.54 (d, *J* = 4.3, 1H), 5.17 (s, 2H), 5.49 (d, *J* = 4.3, 1H), 7.33–7.44 (m, 5H); ^13^C-NMR (75 MHz, CDCl_3_): δ 177.7, 168.0, 134.8, 128.7, 69.9, 67.4, 64.0, 62.8, 31.7, 27.0.

#### 3.1.7. General Procedure for the Synthesis of **11a**–**d**

DCC (0.33 mmol) was added to a solution of the acid **6** (0.30 mmol) in anhydrous dichloromethane (3.8 mL) at room temperature and stirred for 30 min. After filtration on Celite, **10** was added to the solution and the reaction mixture was stirred overnight at room temperature. The solvent was then evaporated to dryness under vacuum [33]. The residue obtained was purified by chromatography on silica gel using dichloromethane/AcOEt (9/1 to 7/3) as eluent to give **11** as inseparable mixture of diastereomers.

*(2S,5R,6R)-Benzyl 6-(2-(2-oxo-1,4-diphenylazetidin-3-yl)acetamido)-3,3-dimethyl-7-oxo-4-thia-1-aza-bicyclo[3.2.0]heptane-2-carboxylate* (**11a**). Colorless oil isolated as inseparable mixture of diastereomers (55% yield). IR (neat, cm^−1^): ν 3320, 3047, 1746, 1676, 1501, 1461, 1389; ^1^H-NMR (250 MHz, CDCl_3_): δ 1.39 (s, 3H), 1.61 (s, 3H), 2.81 (dd, *J* = 8.0, 15.8, 1H), 2.92 (dd, *J* = 7.0, 15.8, 1H), 3.34–3.40 (m, 1H), 4.47 (s, 1H), 4.84 (d, *J* = 2.2, 1H ), 4.87 (d, *J* = 2.2, 1H), 5.18 (s, 2H), 5.51 ( d, *J* = 4.2, 1H), 5.54 (d, *J* = 4.2, 1H), 5.61–5.69 (m, 1H), 7.01–7.37 (m, 15H); ^13^C-NMR (62.89 MHz, CDCl_3_): δ173.4, 169.3, 167.5, 166.6, 137.2, 136.9, 136.8, 134.7, 129.1, 128.7, 128.6, 126.0, 124.2, 117.2, 70.3, 68.0, 67.5, 64.9, 60.6, 60.4, 59.1, 58.9, 55.5, 32.0, 26.8, 21.0, 14.2 MS: *m/z* 570 (M^+^). Anal. calcd. for C_32_H_31_N_3_O_5_S: C, 67.47; H, 5.48; N, 7.38%; found: C, 67.44; H, 5.50, N, 7.36%.

*(2S,5R,6R)-Benzyl 6-(2-(1-(3,4-dimethylphenyl)-2-oxo-4-phenylazetidin-3-yl)acetamido)-3,3-dimethyl-7-oxo-4-thia-1-aza-bicyclo[3.2.0]heptane-2-carboxylate* (**11b**). Yellow oil isolated as inseparable mixture of diastereomers (69% yield). IR (neat, cm^−1^): ν 3463, 2958, 1746, 1671, 1508, 1463, 1394; ^1^H-NMR (300 MHz, CDCl_3_): δ 1.40 (s, 3H), 1.41 (s, 3H), 1.63 (s, 3H), 1.64 (s, 3H), 2.17 (s, 12H), 2.80 (dd, *J* = 7.1, 15.7, 2H), 2.91 (dd, *J* = 7.1, 15.7, 2H), 3.30–3.37 (m, 2H), 4.47 (s, 2H), 4.79 (d, *J* = 2.3, 1H ), 4.82 (d, *J* = 2.3, 1H), 5.19 (s, 4H), 5.52 (d, *J* = 4.2, 1H), 5.54 (d, *J* = 4.2, 1H), 5.63 (dd, *J* = 4.2, 8.4, 1H), 5.66 (dd, *J* = 4.2, 8.4, 1H), 6.85–6.98 (m, 1H), 7.15 (m, 4 H), 7.37 (m, 12H); ^13^C-NMR (75 MHz, CDCl_3_): δ 173.4, 169.2, 167.5, 166.2, 166.1, 137.5, 137.1, 135.1, 134.6, 132.7, 129.9, 129.1, 128.7, 125.9, 118.6, 114.4, 70.4, 68.0, 67.4, 64.9, 60.8, 60.4, 59.1, 58.9, 55.3, 32.0, 31.9, 26.8, 19.8, 19.2. MS: *m/z* 620 [M+Na]^+^. Anal. calcd. for C_34_H_35_N_3_O_5_S: C, 68.32; H, 5.90; N, 7.03%;found: C, 68.36; H, 5.84, N, 7.05%.

*(2S,5R,6R)-Benzyl 6-(2-(1-(4-methoxylphenyl)-2-oxo-4-phenylazetidin-3-yl)acetamido)-3,3-dimethyl-7-oxo-4-thia-1-aza-bicyclo[3.2.0]heptane-2-carboxylate* (**11c**). Yellowish oil isolated as inseparable mixture of diastereomers (54% yield). IR (neat, cm^−1^): ν 3379, 2949, 1746, 1681, 1518, 1246; ^1^H-NMR (300 MHz, CDCl_3_): δ 1.40 (s, 6H), 1.63 (s, 6H), 2.80 (dd, *J* = 7.1, 15.7, 2H), 2.91 (dd, *J* = 7.1, 15.7, 2H), 3.30-3.37 (m, 2H), 3.73 (s, 6H), 4.47 (s, 2H), 4.79 (d, *J* = 2.3, 1H ), 4.82 (d, *J* = 2.3, 1H), 5.19 (s, 4H), 5.52 (d, *J* = 4.2, 1H), 5.54 (d, *J* = 4.2, 1H), 5.62–5.69 (m, 2H), 6.77 (d, *J* = 9.0, 4H), 7.21 (d, *J* = 9.0, 4 H), 7.27–7.37 (m, 20H); ^13^C-NMR (100.03 MHz, CDCl_3_): δ 173.6, 169.2, 167.5, 156.2, 141.2, 139.9, 134.6, 130.6, 129.1(×2), 128.7, 126.1 (×3), 118.5 (×2), 114.4 (×2), 70.4, 68.0, 67.5, 65.0, 61.2, 60.7, 59.1, 55.5, 35.4, 34.9, 32.2, 31.5, 26.8 . MS: *m/z* 620 [M+Na]^+^. Anal. calcd. for C_33_H_33_N_3_O_6_S: C: 66.09, H: 5.55, N: 7.01;found: C: 66.06, H: 5.57, N: 7.04.

*(2S,5R,6R)-Benzyl 6-(2-(1-(4-methoxylphenyl)-2-(4-nitrophenyl)-4-oxoazetidin-3-yl)acetamido)-3,3-dimethyl-7-oxo-4-thia-1-aza-bicyclo[3.2.0]heptane-2-carboxylate* (**11d**). Yellowish oil isolated as inseparable mixture of diastereomers (45% yield). IR (neat, cm^−1^): ν 2929, 1751, 1666, 1513, 1349, 1246; ^1^H-NMR (400 MHz, CDCl_3_): δ 1.40 (s, 6H), 1.63 (s, 6H), 2.82 (dd, *J* = 8.0, 16.0, 2H), 2.95 (dd, *J* = 5.2, 16.0, 2H), 3.32-3.36 (m, 2H), 3.74 (s, 6H), 4.47 (s, 2H), 4.95 (d, *J* = 2.0, 1H), 4.98 (d, *J* = 2.0, 1H), 5.18 (s, 4H), 5.50 (d, *J* = 4.4, 1H), 5.55 (d, *J* = 4.4, 1H), 5.60–5.68 (m, 2H), 6.79 (d, *J* = 9.2, 4H), 7.16 (d, *J* = 9.2, 4 H), 7.36–7.39 (m, 10H), 7.55–7.59 (m, 4 H), 8.22 (d, *J* = 8.4, 4H); ^13^C-NMR (100.03 MHz, CDCl_3_): δ 173.2, 169.0, 167.3, 164.8, 156.5, 148.0, 144.6, 138.1, 134.6, 130.3, 128.7, 127.2, 127.0, 124.3, 118.4, 114.5, 70.4, 67.8, 65.1, 60.3, 58.7, 55.4, 32.0, 29.7, 26.9 . MS: *m/z* 644 [M]^+^. Anal. calcd. for C_33_H_32_N_4_O_8_S: C: 61.48, H: 5.00, N: 8.69; found: C: 61.51, H: 4.98, N: 8.72.

#### 3.1.8. General Procedure for the Synthesis of **12a**–**d**

10 mol % Pd/C (46 mg) was added to a solution of **11** (0.039 mmol) in a mixture of tetrahydrofuran (3.9 mL), water (3.9 mL) and 0.006 g (0.08 mmol) of NaHCO_3_. The reaction mixture was left at rt overnight connected to a double layer balloon of hydrogen. The catalyst was filtered and washed with ethanol (4 mL) and water (4 mL). The combined filtrate was extracted with diethyl ether (2 × 8 mL) and the aqueous phase was freeze-dried yielding in high purity the compound **12** as a sodium salt [34].

*Sodium (2S,5R,6R)-6-(2-(2-oxo-1,4-diphenylazetidin-3-yl)acetamido-3,3-dimethyl-7-oxo-4-thia-1-aza-bicyclo[3.2.0]heptane-2-carboxylate* (**12a**). Light gry solid isolated as inseparable mixture of diastereomers (76% yield m.p. 240–243 °C). IR (KBr pellet, cm^−1^): ν 3400, 2924, 1751, 1662, 1592, 1389, 1503; ^1^H-NMR (300 MHz, D_2_O): δ 1.39 (s, 3H), 1.40 (s, 3H), 1.54 (s, 3H), 1.55 (s, 3H), 2.81–3.00 (m, 4H), 3.49–3.57 (m, 2H), 4.18 (s, 2H), 5.03 (d, *J* = 2.5, 1H), 5.07 (d, *J* = 2.5, 1H), 5.34 ( d, *J* = 4.2, 1H), 5.38 (d, *J* = 4.2, 1H), 5.45 (d, *J* = 4.2, 1H), 5.51 (d, *J* = 4.2, 1H), 7.12–7.45 (m, 20H); ^13^C-NMR (100.03 MHz, D_2_O): δ 175.7, 175.3, 173.3, 170.1, 169.5, 137.6, 137.4, 137.1, 130.2, 130.0, 129.6, 127.4, 125.9, 118.8, 74.0, 72.4, 65.4, 65.2, 61.3, 59.0, 31.5, 28.5, 27.2. MS: *m/z* 501 [M+Na]^+^. Anal. calcd. for C_25_H_24_N_3_O_5_SNa: C: 59.87, H: 4.82, N: 8.38; found: C: 59.85, H: 4.80, N: 8.36.

*Sodium (2S,5R,6R)-6-(2-(1-(3,4-Dimethylphenyl)-2-oxo-4-phenylazetidin-3-yl)acetamido)-3,3-dimethyl-7-oxo-4-thia-1-aza-bicyclo[3.2.0]heptane-2-carboxylate* (**12b**). Pearly white solid as inseparable mixture of diastereomers (67% yield. m.p. 235–238 °C). IR (KBr pellet, cm^−1^): ν 3439, 2963, 1751, 1676, 1617, 1508, 1399; ^1^H-NMR (250 MHz, CDCl_3_): δ 1.40 (s, 3H), 1.43 (s, 3H), 1.55 (s, 6H), 2.13(s, 12H), 2.87–2.94 (m, 4H), 3.43–3.52 (s, 2H), 4.19 (s, 2H), 5.03 (d, *J* = 2.8, 1H), 5.07 (d, *J* = 2.8, 1H), 5.34 (d, *J* = 4.0, 1H), 5.37 (d, *J* = 4.0, 1H), 5.46 (d, *J* = 4.0, 1H), 5.51 (d, *J* = 4.0, 1H), 6.98–7.10 (m, 6 H), 7.35–7.43 (m, 10H); ^13^C-NMR (100.03 MHz, D_2_O): δ 174.9, 172.3, 172.1,168.1, 137.7, 137.6, 135.4, 132.9, 130.4, 129.4, 129.0, 126.7, 119.5, 119.1, 115.9, 115.3, 74.1, 67.2, 64.9, 60.7, 58.6, 56.5, 55.6, 34.1, 31.0, 30.7, 27.5, 27.3, 19.8, 19.1. MS: *m/z* 529 [M + Na]^+^. Anal. Calcd. for C_27_H_28_N_3_ O_5_SNa: C: 61.23, H: 5.33, N: 7.93;found: C: 61.26, H: 5.34, N: 7.91.

*Sodium (2S,5R,6R)-6-(2-(1-(4-methoxyphenyl)-2-oxo-4-phenylazetidin-3-yl)acetamido)-3,3-dimethyl-7-oxo-4-thia-1-aza-bicyclo[3.2.0]heptane-2-carboxylate* (**12c**). Yellow-white solid as inseparable mixture of diastereomers (80% yield, m.p. 245–248 °C). IR (KBr pellet, cm^−1^): ν 3439, 2934, 1741, 1657, 1602, 1518, 1399, 1251; ^1^H-NMR (600 MHz, D_2_O): δ 1.44 (s, 3H), 1.48 (s, 3H), 1.58 (s, 3H), 1.59 (s, 3H), 2.89 (dd, *J* = 9.6, 15.0, 2H), 2.96–3.00 (m, 2H), 3.50–3.58 (m, 2H), 3.77 (s, 6H), 4.23 (s, 2H), 5.08 (d, *J* = 2.4, 1H), 5.11 (d, *J* = 2.4, 1H), 5.38 (d, *J* = 3.6, 1H), 5.41 (d, *J* = 4.3, 1H), 5.50 (d, *J* = 3.6, 1H), 5.55 (d, *J* = 4.3, 1H), 6.94 (d, *J* = 7.4, 4H), 7.27 (d, *J* = 7.4, 4H), 7.42–7.48 (m, 10H) ); ^13^C-NMR (151 MHz, D_2_O): δ 175.4, 175.1, 174.7, 173.4, 172.9, 168.7, 155.9, 144.5, 137.2, 130.7, 129.9, 129.0, 128.8, 126.4, 119.6, 119.4, 114.5, 79.2, 76.7, 73.8, 73.0, 67.3, 67.1, 66.9, 65.1, 64.4, 61.5, 61.3, 60.7, 58.9, 58.0, 56.3, 55.4, 30.4, 28.6, 28.3, 27.1, 27.0. MS: *m/z* 531 [M + Na]^+^. Anal. Calcd. for C_26_H_26_N_3_ O_6_SNa: C: 58.75, H: 4.93, N: 7.91; found: C: 58.73, H: 4.90, N: 7.92.

*Sodium (2S,5R,6R)-6-(2-(1-(4-Methoxyphenyl)-2-(4-nitrophenyl)-4-oxo-azetidin-3-yl)acetamido)-3,3-dimethyl-7-oxo-4-thia-1-aza-bicyclo[3.2.0]heptane-2-carboxylate* (**12d**). Dark brown solid as inseparable mixture of diastereomers (68% yield, m.p. 123–125 °C). IR (KBr pellet, cm^−1^): ν 3443, 2964, 2919, 1736, 1657, 1612, 1513, 1399, 1384, 1251; ^1^H-NMR (400 MHz, D_2_O): δ 1.44 (s, 3H), 1.49 (s, 3H), 1.56 (s, 3H), 1.58 (s, 3H), 2.85–3.00 (m, 4H), 3.37–3.51 (m, 2H), 3.64 (s, 3H), 3.65 (s, 3H), 4.22 (s, 2H), 4.95–5.01 (m, 2H), 5.35 (d, *J* = 2.4, 1H), 5.40 (d, *J* = 2.4, 1H), 5.47 (d, *J* = 3.6, 1H), 5.54 (d, *J* = 3.6, 1H), 6.72–7.21 (m, 16 H); ^13^C-NMR (100 MHz, D_2_O):δ 174.8, 172.8,168.6, 157.1, 156.19, 143.3, 145.1, 130.3, 128.3, 137.3, 124.9, 124.4, 120.1, 19.1, 116.9, 114.9, 114.6, 77.5, 66.9, 66.8, 64.7, 60.9, 58.5, 55.8, 46.4, 37.6, 30.9, 26.7; MS: *m*/*z* 576 [M + Na]^+^. Anal. calcd. for C_26_H_25_N_4_ O_8_SNa: C: 54.16, H: 4.37, N: 9.72; found: C: 54.13, H: 4.34, N: 9.76.

### 3.2. Microbiological Assays and Bacterial Strains

The minimum inhibitory concentrations (MICs) of the different antibiotics was estimated by means of the broth microdilution method, using the densities (5 × 10^5^ CFU·mL^−1^) and protocolsrecommendedby the Clinical and Laboratory Standards Institute (CLSI), formerly the National Committee for Clinical Laboratory Standards (NCCLS) [35]. Slight modifications were the incubation at 37 °C and shaking at 200 rpm of the bacterial cultures. The effects of the different antibiotics concentrations on the microbial growth was evaluated by turbidity, by measuring optical density at 600 nm (OD_600_). MIC_100_ was defined as the minimum antibiotic concentration that does not change the sample turbidity compared to time 0, while the MIC_50_ as the minimal concentration that reduced OD_600_ of 50% compared to that ofinoculated sample without antibiotic. Minimum lethal dose (MLD) was estimated by CFU·mL^−1^ determination. Briefly, for each examined antibiotic, different dilutions of each bacterial inoculum growth for 24 h in the presence of antibiotic concentrations ≥ MIC_100_ were spread on plate count agar (PCA) and CFU were counted after incubation 24 h at 37 °C. The MLD was the minimum antibiotic concentrationat which the number of CFU·mL^−1^ resulted equal to 0.

The *in vitro* antibacterial activity of the new compound was compared to that of the ampicillin (Sigma-Aldrich), a reference beta-lactam agent in clinical use for the treatment of infections caused from Gram positive (*Streptococcus* spp., *Staphyloccus* spp.) and few Gram negative bacteria (*Neisseria*, some *Enterobacteriaceae*).

Bacterial strain used for *in vitro* antibiotic activity determination included patogenic and not patogenic clinical and environmental isolated, both Gram negative *Escherichia coli*, *Salmonella*, *Pseudomonas fluorescens*, *Pseudomonas aeruginosa* and Gram positive *Staphylococcus aureus*, *Staphylococcus* *epidermidis*, *Bacillus* sp. *Escherichia coli* (strain JM109) was purchased from Promega [36]. *Salmonella enterica* subsp*. enterica* serovar *thyphimurium,* strain LT2 ATCC 700720 was purchased from Leibniz Institute DSMZ-German Collection of Microorganisms and Cell Culture [37]. The remaining strains were derived from the collection deposited in the microbiology laboratory directed by G. Vigliotta.

### 3.3. In vitro NIH-3T3 Cells Line Cytotoxicity Testing

#### 3.3.1. Cell Cultures

NIH-3T3 cells (murine fibroblast) were obtained from ATCC (American Type Culture Collection, Rockville, MD, USA). Cells were grown in DMEM medium with heat-inactivated 10% FCS, 100 mg·mL^−1^ penicillin, 100 mg·mL^−1^ streptomycin, by incubating in a humidified atmosphere of 95% air/5% CO_2_ at 37 °C.

#### 3.3.2. Cell Viability Assay

The cytotoxicity of the synthesized compounds was detectedby the mitochondrial cytotoxic test, using thiazolyl blue tetrazolium bromide (MTT) as an indicator of metabolically active cells. The assay was performed in 96-well microplates. NIH-3T3 cells were seeded at a density of 1.0 × 10^5^ cells per well and growth 24 h at 37 °C before the addition of the compounds. Antibiotics were dissolved in 50% DMSO (*v*/*v*, in water) and subsequently added to the cells to the final concentration of 2 μM to 50 μM (final concentration of DMSO 0.5%). Microplates were incubated for 72 h at 37 °C. After incubation thiazolyl blue tetrazolium bromide (3.33 mg·mL^−1^ phosphate buffered saline, pH = 7.4) was added to each well and left to incubate for further 4 h to allow for intracellular reduction of the soluble yellow MTT to the insoluble purple formazan dye. Then the medium with MTT solution was removed. Formazan crystals in viable cells were dissolved in the lysis solution (4 M MHCl and 0.1% Nonidet P40 in ethanol) and absorbance was measured at 540 nm. As a control, the same cells were exposed to 0.5% DMSO alone and inhibition activity of compounds was expressed as percentages of control. Each value is the mean of 6 wells with standard deviation.

## 4. Conclusions

In summary, the present work describes a new and easy protocol for the synthesis of some novel semi-synthetic penicillin analogues by the conjunction of substituted β-lactam nucleus with a penicillanic acid framework. The involvement in the synthetic route of a Staudinger reaction constitutes a convenient approach for the functionalization of the intermediate β-lactam units and consequently for the introduction of different substituents on the target scaffold. All target compounds were screened for their antibacterial activities, and from the biological evaluation it appeared that the introduction of a β-lactam moiety at the amino end of 6-amino penicillanic acid positively affected the antibacterial ability. In general, all target compounds exhibited potent antibacterial activity against Gram positive bacteria in most of the cases higher than that of reference antibiotic ampicillin. Further, some title compounds were also assessed for their cytotoxic activity against NIH-3T3 cell lines using MTT assay method. The cytotoxic screening displayed that these compounds did not cause any cytotoxic effect at the concentrations higher than that required to exert antibiotic activity. The promising antimicrobial activity and low-toxicity profile of targets make them promising molecules for further studies. The development of new derivatives and the investigations devoted to the mode of antibacterial action of these new compounds are now underway.
